# Characteristics of disability in activity of daily living in elderly people associated with locomotive disorders

**DOI:** 10.1186/s12877-017-0543-z

**Published:** 2017-07-26

**Authors:** Tsutomu Iwaya, Tokuhide Doi, Atsushi Seichi, Yuichi Hoshino, Toru Ogata, Masami Akai

**Affiliations:** 1 Nagano University of Health and Medicine, 11-1 Imaihara Kawanajima-chou Nagano-shi, Nagano, 381-2227 Japan; 2 Geriatric Care Facilities Excellent Care Shizu, 1316-1 Kami-Shizu, Sakura, Chiba, 285-0846 Japan; 3 Mitsui Memorial Hospital, 1 Kanda-Izumi-chou, Chiyoda-ku, Tokyo, 101-0024 Japan; 4 Tochigi Rehabilitation Center, 3337-1 Komanyu-machi, Utsunomiya, Tochigi, 320-8503 Japan; 5 National Rehabilitation Center for Persons with Disabilities, 4-1 Namiki, Tokorozawa, Saitama, 359-8555 Japan; 6 International University of Health and Welfare, 4F, Aoyama 1-Chome Tower, 1-3-3 Minami-Aoyama, Minato-ku, Tokyo, 107-0062 Japan

**Keywords:** Locomotive syndrome, Geriatric Locomotive Function Scale-25, Activity limitation, Degree of disability, Disablement process

## Abstract

**Background:**

Ageing is associated with a decline of motor function and ability to perform daily activities. Locomotive disorders are one of the major disorders resulting in adverse health condition in elderly people. Concept of Locomotive syndrome (LoS) was proposed to tackle the problems and prolong healthy life expectancy of people with locomotive disorders. To develop intervention strategy for LoS it is mandatory to investigate impairments, functional disabilities which people with locomotive disorder experience and to examine relationships among these parameters. For this purpose we have developed Geriatric Locomotive Function Scale-25 (GLFS-25). Though several physical performance tests were reported for identification or monitoring the severity of LoS, there are few studies reported on characteristics of disability which people with locomotive disorders experience.

The aim of this study was to report the characteristics of ADL disabilities in elderly people with locomotive disorders in terms of numbers and degree of activity limitations.

**Methods:**

We organized a cohort study and recruited 314 participants aged 65 years and over from five orthopedic clinics or nursing care facilities. This was a cross-sectional study to use the baseline data of such cohort. ADL disabilities were assessed using GLFS-25 scale arranging the GLFS-25 scores in ordinal levels using “R language” program. Numbers and degrees of activity limitations were determined and compared among the levels. Frequency of limitation in activities regarding social activity, housework, locomotion, mobility and self-care was compared among across the disability level.

**Results:**

The GLFS-25 score was mathematically categorized into 7 levels. The number of activity limitations and the degrees of each activity limitation were significantly greater in high GLFS-25 levels than in low levels. Difficulties in mobility appeared in less severe level, difficulties in domestic and social life appeared in moderately severe level, and difficulties in self-care appeared in advanced level.

**Conclusions:**

High GLFS-25 score represented high degree of disability on ADLs. Concordant increase of numbers of activity limitation and severity progression in activity limitation may contribute to progression of disability. Activity limitation may occur in the following order: sports activity, walking, transferring, and self-care.

## Background

Aged society is accelerating rapidly in Japan. The total population of Japan was 127.11 million and the number of people aged 65 and over was 33.92 million as of October 1, 2015. The ratio of elderly people aged 65 and over was 26.7% [1] and it is expected to extend 30% in 2025 and reach 39.9% in 2060 [2]. The number of persons needing long-term care is increasing rapidly among elderly people, and the number of people more than 65 years old who are certified as requiring long-term care is 5.84 million (4.6% of total population) at the end of fiscal year 2013 [3]. Many elderly people want to continue to live in their own houses and receive long term care [4]. As elderly people suffer from many diseases and experience disability in activities of daily living (ADL) due to declined functional limitations, it is necessary for health care professionals to provide interventions not only to control diseases, but to restore or maintain physical function and prevent or reduce disability.

Recently growing and important cause of the condition requiring Long Term Care Insurance (LTCI) support was locomotive organ disorders. The percentage of people utilizing LTCI services due to locomotive organ disorders (falls/fractures and joint disorders) has increased from 21.1% in 2010 [5] to 22.7% in 2013 [6].

The concept of “Locomotive syndrome (LoS)” was proposed to tackle such problems and to prolong healthy life expectancy of people with locomotive disorders. This syndrome refers to those elderly people who have come to need care services because of problems of the locomotive organs, or have risk conditions which may require them to have such services in the future [7, 8]. The total number of individuals with LoS between the 40s and 70s in Japan was estimated to be approximately 7.5 million [9]. To prolong healthy life expectancy of the elderly people under the condition of LoS, it is necessary to develop intervention strategy for those who are currently healthy but at risk of disabling due to declining locomotor function [10] as well as for those who are disabled due to locomotor dysfunction and need nursing care. This concept does not depend upon traditional pathological basis. Actual pathological changes cover multiple organs derived from degenerative process. The Japanese Orthopedic Association started campaign to raise public awareness on interest in locomotive organ and facilitate early detection of those who are at risk of requiring support [11].

To identify people under the condition of LoS self-reported questionnaire on ADL disabilities (GLFS-25) [12] and motor function tests were developed [13]. Recent studies reported factors concerning pathology, impairments and functional limitations to identify people at risk of LoS. Osteoporosis, pain on low back, knee [14, 15], obesity [16], and spinal deformity [17] were reported to be risk factors for LoS. Several physical performance tests such as timed up and go test, one-leg standing time, back muscle strength, leg extension power, gait speed, grip power, and maximum stride, were proved to be valid for identification of LoS [18–20]. In all of these studies, LoS was detected by the Geriatric Locomotive Function Scale (GLFS) -25 total score.

A few studies were reported concerning types and degree of the ADL disabilities among elderly people with locomotive organ disorders. Tobimatsu [21] suggested that people developed difficulties in IADL items earlier than in ADL items. Moreover, mild difficulties in going up- and downstairs, walking briskly and long-distance walking (more than 2–3 km), along with body pain (upper/lower extremities, back or neck), were experienced before the deficits in IADL or social functions were noted. Iwaya, Akai, and Doi [20] also reported that the number of difficult daily tasks increased in accordance with aggravation of locomotive disability.

To develop appropriate therapeutic regimen tailored to individuals with different severity of disability, it is necessary to investigate disablement process that include which factors are relevant to aggravation of disability and how the degree of disability progress. Severity of ADL disability is related to determining care needs of LTCI System. Support/care level of LTCI may differ from assigned care level based on the estimated care minutes due to personal particular needs combining living condition, family support, and other factors [22]. Considering the limitation of LTCI grading system, which is not purely limited in the degrees of support/care needs, it is necessary to investigate the degree of disability using measure that is valid for medical practice and research.

The Geriatric Locomotive Function Scale-25 (GLFS-25) was originally developed to screen the persons with locomotive dysfunction. GLFS-25 included 4 questions regarding pain, 19 ADL, and 2 anxiety (Table 1) [12]. Seichi et al. [9] reported age specific standard values on the GLFS-25 which was 4.4 in the 40s, 5.5 in the 50s, 7.1 in the 60s, and 12.7 in the 70s.

**Table 1:** Geriatric Locomotive Function Scale (GLFS) -25

Please answer on your status over the last one month		
Question items	Domain	Response options
1. Did you have any pain (including numbness) in your neck or upper limbs (shoulder, arm, or hand)?	pain	0: no pain 1: mild pain 2: moderate pain 3: considerable pain 4: severe pain
2. Did you have any pain in your back, lower back or buttocks?	pain	
3. Did you have any pain (including numbness) in your lower limbs (hip, thigh, knee, calf, shin, ankle, or foot)?	pain	
4. To what extent has it been painful to move your body in daily life?	pain	
**5.** To what extent has it been difficult to get up from a bed or lie down?	mobility	0: not difficult 1: mildly difficult 2: moderately difficult 3: considerably difficult 4: extremely difficult
**6.** To what extent has it been difficult to stand up from a chair?	mobility	
**7.** To what extent has it been difficult to walk inside the house?	mobility	
**8.** To what extent has it been difficult to put on and take off shirts?	self-care	
**9.** To what extent has it been difficult to put on and take off trousers and pants?	self-care	
**10.** To what extent has it been difficult to use the toilet?	self-care	
**11.** To what extent has it been difficult to wash your body in the bath?	self-care	
**12.** To what extent has it been difficult to go up and down stairs?	mobility	
**13.** To what extent has it been difficult to walk briskly?	mobility	
**14.** To what extent has it been difficult to keep yourself neat?	self-care	
**15.** How far can you keep walking without rest? (please select the closest answer)	mobility	0: more than 2-3 km 1: approximately 1 km 2: approximately 300 m 3: approximately 100 m 4: approximately 10 m
**16.** To what extent has it been difficult to go out to visit neighbors?	Interpersonal interactions	0: not difficult 1: mildly difficult 2: moderately difficult 3: considerably difficult 4: extremely difficult
**17.** To what extent has it been difficult to carry objects weighing approximately 2 kg (2 standard milk bottles or 2 PET bottles each containing 1 l)?	mobility	
**18.** To what extent has it been difficult to go out using public transportation?	mobility	
**19.** To what extent have simple tasks and housework (preparing meals, cleaning up, etc.) been difficult?	Domestic life	
**20.** To what extent have load-bearing tasks and housework (cleaning the yard, carrying heavy bedding, etc.) been difficult?	Domestic life	
**21.** To what extent has it been difficult to perform sports activity (jogging, swimming, gate ball, dancing, etc.)?	Community, social life	
**22.** Have you been restricted from meeting your friends?	Interpersonal interactions	0: not restricted 1: slightly restricted 2: restricted about half the time 3: considerably restricted 4: gave up all activities
**23.** Have you been restricted from joining social activities (meeting friends, playing sport, engaging in activities and hobbies, etc.)?	Community, social life	
24. Have you ever felt anxious about falls in your house?	anxiety	0: have not felt anxious 1: have occasionally felt anxious 2: have sometimes felt anxious 3: have often felt anxious 4: have constantly felt anxious
25. Have you ever felt anxious about being unable to walk in the future?	anxiety	

The question items in boldface are the daily activities selected in analysis of the frequency of poor response

From 25 question items, 4 regarding body pain and 2 regarding anxious feeling are omitted, and remaining 19 items provide response ratio (in bold letters)

(This table is reused from our paper in Quality of Life Research under permission)

The validity of GLFS-25 was confirmed by demonstrating a significant correlation and association of its score with the outcome of a series of functional performance tests [18, 20]. Grade of physician-judged locomotive dysfunction was significantly related to GLFS-25 score [23].

According to these previous reports, we considered GLFS-25 was valid to investigate patterns of ADL disability in persons with locomotive disorders. We used 19 questions items regarding difficulties doing basic (mobility and personal care) and instrumental (household management and social life) ADLs to clarify the order of difficulties doing daily activities.

The aim of this study was to investigate the factors related to grades of ADL disability, and identify activity limitations relevant to the individual levels of ADL disability.

## Methods

We used the data collected in a prospective cohort study on the disablement process of locomotive disability and development of guideline for locomotive disability prevention (“LDP study supported by a Sciences Research Grant from the Ministry of Health, Labor and Welfare, Japan (H21-Choju-G006)”). The participants joined outpatient rehabilitation programs at 5 facilities and were examined 4 times: at baseline and after 6, 12, and 18 months. We used the baseline data at the initial survey in this study. The detail of data collection for this cohort study was the same as that previously reported [24, 25].

### Participants

Patients aged ≥65 years (*N* = 314) were recruited from 5 orthopedic clinics and affiliated nursing care facilities which were located in 3 urban (Tokyo, Hamamatsu-shi, Hiroshima-shi) and 2 rural (Aizu Wakamatsu-shi, Nakatsu-shi) area. Written informed consent was obtained from all participants.

### Inclusion criteria

1. Age ≥ 65 years (either gender)
2. Any 1 of the following 4 criteria
  1. Complaints related to the legs or spine without disability in walking or leaving the home (outpatients).
  2. Complaints related to the legs and spine, and slight disabilities in walking and leaving the home (outpatients).
  3. Slight disability in walking due to locomotive organ disorders (users of long-term care services).
  4. Complaints related to the upper extremities without disability in walking or leaving the home (outpatients at orthopedic clinics).
3. Ability to answer the GLFS- 25 questionnaire without assistance.
4. Consent to radiographic examination of the knees and spine.
5. Consent to examination of the serum vitamin D and hyaluronic acid levels.
6. Consent to participate in the following motor function tests: one-leg standing test, measurements of grip power and leg extension power, 100-step test, and trunk forward bending test.

### Exclusion criteria

1. Inability to stand up from a chair or bed.
2. Disability in walking or locomotion because of neurological disease requiring admission treatment.
3. Severe pulmonary, renal, coronary, or hepatic disease.
4. Mental illness.
5. Past history of stroke within the preceding 6 months.
6. Past history of myocardial infarction within the preceding 6 months.
7. Past history of fracture of a lower extremity within the preceding 6 months.
8. Current treatment for acute trauma.
9. Other reasons determined by the attending physician.

### Assessments

Participants were asked about his or her history of falls and fractures, regular medications, diagnoses related to the locomotive organs, comorbidities, use of walking aids, living environment (especially number of family members needed for care), and physiotherapeutic interventions, and requested each participant to complete the GLFS-25 questionnaire.

The attending medical staff examined the patient based on the complaints and the specific painful area (back, buttock, posterior thigh, and knee), and recorded the physical findings related to the trunk and lower extremities. They also measured the body height, body weight, range of motion (ROM) of the hip and knee joints, and strength of the iliopsoas, quadriceps, anterior tibialis, and calf muscles, and recorded the results of the motor function tests, including the one-leg standing time, grip power, leg extension power, 100-step test, and trunk forward bending distance.

The staff obtained radiographs, including an anteroposterior view of the knee joints in a standing posture and a lateral view of the thoracolumbar spine, and assessed them quantitatively using semi-automated computer-aided diagnosis. The bone density of the wrist or metacarpal bones, the lumbar spine, or the proximal femur was measured using X-ray absorptiometry (DEXA), digital image processing (DIP), or quantitative computed tomography (QCT), and expressed as the percentage of the mean for young adults (YAM). Serum vitamin D and hyaluronic acid levels were also examined.

### Scale for assessing ADL ability: Geriatric Locomotive Function Scale-25

The Geriatric Locomotive Function Scale-25 (GLFS-25) was originally developed to screen the persons with locomotive dysfunction. GLFS-25 included 4 questions regarding pain, 5 questions regarding self-care (put on and take off shirts and pants, wash body, use toilet and keep oneself neat), 8 questions regarding mobility (get up from bed and lie down, stand up from a chair, walk inside the home, go up and down stairs, walk briskly, keep walking, carry heavy object and use public transportation), 2 domestic life (simple housework and heavy housework), 2 community social life (join sports activity and social activity), 2 interpersonal interaction (visit neighbors and friends), and 2 anxiety (anxious about fall and inability of walk). Response choices to these 25 items are graded with 5-point scales: no impairment (0 points: Not difficult to do), mild impairment (1 points: mildly difficult to do), moderate impairment (2 points: moderately difficult to do), considerable impairment (3 points: considerably difficult to do) and severe impairment (4 points: extremely difficult to do) and then arithmetically added to produce a total score (minimum 0, maximum 100) (Table 1). The cut-off score for identifying Los was set at 16 on GLFS-25 total score [12]. Participants were asked to answer the GLFS-25 questions by themselves.

Responses to 19 items except pain and anxiety regarding daily activities were used to identify factors related to the severity of ADL disability and activity limitations relevant to the levels of ADL disability.

### Definition of poor responses, poor response ratio

If participants reported no difficulty to do on a specific GLFS-25 question item, we considered the participant experienced no limitation in the activity represented by the question item. If participants reported mild, moderate, considerable or extreme difficulty (PR: Poor Response) to do on a certain GLFS-25 question item, we considered the participant experienced activity limitation in the activity represented by the question item. Severity of activity limitation was assessed by response grades to GLFS-25 question items. If participants reported difficulties in more than one activity, we considered the participant experienced ADL disability. Poor Response Ratio (PRR) was calculated dividing the total number of group member by number of members who reported PR within each level of ADL disability.

### Analytic methods

1. Classification of level of ADL disability.
  We converted the GLFS-25 scores into ordinal levels using “R language” program for optimal classification of histogram [26, 27], and designated the levels as the grades of ADL disabilities. ‘R’ is a free software programming language for statistical computing and graphics, and it is widely used among statisticians and data miners for statistical software development and data analysis.
2. Associations between numbers of activity limitations per participant and GLFS-25 levels
  To determine grades of ADL disability which participants experience, numbers of PRs (that is activity limitations) per person were compared among GLFS-25 levels using Kruskal-Wallis test followed by multiple comparison test by Bonferroni’s correction. Level of significance was set at <0.05.
3. Associations between the GLFS-25 levels and severity of activity limitations
  Jonckheere-Terpstra tests [28] were used to determine if there were statistically significant trend between the GLFS-25 levels on grade of responses to individual questions.
4. Frequency of activity limitation by the GLFS-25 level
  Poor response ratio for 19 daily activities was calculated within individual GLFS-25 levels in order to identify activity limitations which characterize ADL disability levels.

### Statistical analyses

SPSS version 21 software (IBM, Chicago, IL, USA) was used for all statistical analyses. As variables showed non-normal distribution, statistical analyses to test differences were performed using chi-square test and Kruskal-Wallis test. Multiple comparison tests were performed using Bonferroni’s correction. Significant levels were set at *p* < 0.05 for KW tests and *p* < 0.0023 for Bonferroni’s tests. Jockheere-Terpstra test was used to determine if there were statistically significant trend between GLFS-25 levels on differences of responses.

## Results

### Descriptive characteristics of the participants

Descriptive characteristics were shown in Table 2. Participants were 314 in number (80 men and 234 women) whose ages ranged from 65 to 93 years. The mean age was 75.9 years (Standard deviation SD: 6.3) in the men and 77.9 years (SD: 6.3) in the women. Mean of the GLFS-25 scores was 23.0 (SD: 15.8) ranged from 0 to 73. Numbers of physicians-diagnosed diseases for participants were as follows: knee osteoarthritis was diagnosed for 136 persons; osteoporosis 67, spinal canal stenosis 58, spinal spondylitis 54, and 133 participants had multiple diagnoses. Among this participant group, 268 participants had comorbidity, such as hypertension or diabetes, and 135 participants had multiple comorbidities. The number of participants with low back pain was 212, with gluteal pain 94, with sciatic pain 44, and with knee joint pain 203. The mean grip power was 20.8 kg (SD: 6.9), mean one-leg standing time was 17.5 s (SD: 19.6). The mean serum hyaluronic acid was 123.1 ng/ml (SD: 91.6), mean serum 25OH vitamin D was 44.7 ng/ml (SD: 22.2), mean bone mineral density was 78.2% YAM (SD: 17.8). One hundred and forty-three participants used walking aids, such as a stick or walkers. A history of falling was reported by 233 participants, and 158 had a history of fracture within the past few years.

**Table 2:** Descriptive characteristics of participants

Gender		Male	Female	Total
		80	234	314
Age	mean	75.9	77.9	77.4
	SD	6.3	6.3	6.4
	65–69	19	33	52
	70–74	21	54	75
	75–79	17	56	73
	80–84	18	65	83
	85-	5	26	31
Physicians-diagnosed locomotive disorders				
	Knee Osteoarthritis	28	108	136
	Osteoporosis	0	67	67
	Spinal Canal Stenosis	25	33	58
	Degenerative Spondylosis	9	45	54
	Low Back Pain	9	27	36
	Joint diseases	11	16	27
	vertebral fracture	1	18	19
	Lower Limb fracture	3	15	18
	Hip Osteoarthritis	0	10	10
	Upper Limb fracture	1	8	9
	Others	23	33	56
Number of locomotive disorders per participant				
	1	49	112	161
	2	19	67	86
	3<	7	40	47
Comorbidity				
	Hypertension	47	127	174
	Disorder of lipid metabolism	9	45	54
	Cataract	9	37	46
	Diabetes Mellitus	15	29	44
	Cardiovascular diseases	11	33	44
	Bronchial Asthma	4	7	11
	Others	35	64	99
Number of comorbidities per participant				
	0	9	37	46
	1	33	100	133
	2	22	62	84
	≧3	16	35	51
Functional performance tests	mean	SD	range	median
Leg extension power (kg)	51.7	29.5	3.5–177.5	47.5
One leg standing time with eyes open (sec)	17.5	19.6	0–60	9.1
Grip power (kg)	20.8	6.9	6.0–44.0	20.0
100 steps time (sec)	58.2	11.8	36.0–128.0	56.0
Trunk forward bending distance (cm)	27.1	9.6	3.0–52.5	28.5
Laboratory findings	mean	SD	range	median
Lumbosacral Angle (degree)	30.9	19.1	−37.9 - 73.1	32.4
Femorotibial Angle (degree)	179.1	4.8	166.9–198.4	178.3
Bone density; %YAM	78.2	17.5	40.0–166.0	77.0
Hyaluronic Acid (ng/ml)	124.6	103.1	15.2–800.0	95.9
Vitamin D;25-OH D (ng/ml)	46.1	22.4	7.0–145.0	44.0
Numbers of participants with pain locations			N	Prevalence (%)
	low back pain		212	76.5
	gluteal pain		94	27.3
	sciatic pain		44	14.9
	knee pain		203	64.6
Numbers of participants with limited joint movement			N	Prevalence (%)
	hip		52	16.5
	knee		102	32.5
Number of participants whose muscles strength >MMT 4			N	Prevalence (%)
	iliopsoas		220	70.1
	quadriceps		157	50.0
	anterior tibialis		87	27.7
Numbers of participant who received therapeutic exercise (with overlapping)				
	therapeutic exercise			184
	muscle strengthening exercise		trunk muscles	30
			quadriceps	97
			mixed	35
	balance training			17
	stretching			78
	other exercises			86

Gender, age, diagnosis including comorbidity, results of functional performance tests and laboratory findings, signs and symptoms in lower extremities, and received therapeutic exercise are briefly summarized

### Classification of severity of ADL disability

Using R language program, the GLFS-25 scores were stratified into seven groups as follows: <6, level 1; 7–15, level 2; 16–22, level 3; 23–32, level 4; 33–40, level 5; 41–49, level 6; and ≥50, level 7. The number of the participants classified in the GLFS-25 level 1 was 36, level 2 was 87, level 3 was 68, level 4 was 39, level 5 was 31, level 6 was 27 and level 7 was 22.

### Association between numbers of activity limitation per participant and GLFS-25 levels

Numbers of activity limitation per participant were determined by counting the number of PR. Mean number of activity limitation per participant was 9.1 (SD: 5.4. range: 0–19) for the whole.

**Fig. 1:**
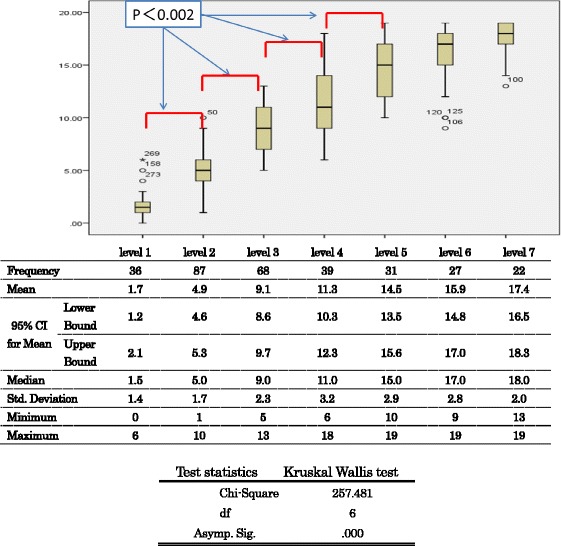
Number of activity limitations of the GLFS-25 scores per participants by 7 levels. Test statistics on differences of item scores among the GLFS-25 total score levels. Kruskal Wallis tests and multiple comparison tests (Bonferroni’s correction) (level of significance KW tests: p<0.05, Multiple comparison: p<0.0023). Participants in low GLFS-25 levels experienced smaller number of activity limitations than in higher GLFS-25 levels

Mean number of activity limitations per participant were 1.7 (SD: 1.4) for level 1 participants, 4.9 (SD: 1.7) for level 2, 9.1 (SD: 2.3) for level 3, 11.3(SD: 3.2) for level 4, 14.5 (SD: 2.9) for level 5, 15.9 (SD: 2.8) for level 6 and 17.4 (SD: 2.0) for level 7. The number of activity limitations was significantly related to the GLFS-25 levels (KW test: *p* < 0.002) (Fig. 1).

Multiple comparison tests revealed significant differences between level 1 and 2, level 2 and 3, level 3 and 4, and level 4 and 5 (Bonferroni’s correction: level of significance *p* < 0.0028).

Table 3 showed mean numbers of individual response item per participant by GLFS-25 levels. The number of response ‘not difficult’ decreased in accordance with progression of ADL disability level. The number of ‘mildly difficult’ increased in accordance with progression of ADL disability levels 1–5, and was the greatest for the level 5 participants. The number of ‘moderately difficult’, ‘considerably difficult’, and ‘extremely difficult’ were increased in accordance with progression of ADL disability level.

**Table 3:** Mean numbers of response items per participant by the GLFS-25 levels from 19 questions

	Level 1	Level 2	Level 3	Level 4	Level 5	Level 6	Level 7	Total
Not difficult	17.3	14.1	9.9	7.7	4.5	3.1	1.6	9.8
Mildly difficult	1.6	3.7	6.3	6.2	7.3	6.8	4.0	4.9
Moderately difficult	0.1	0.7	1.8	2.6	3.3	3.6	4.5	1.9
Considerably difficult	0.0	0.3	0.7	1.6	1.6	3.2	4.2	1.3
Extremely difficult	0.0	0.1	0.3	0.9	1.4	2.3	4.7	0.9

The number of response ‘not difficult’ decreased and the number of ‘moderately difficult’, ‘considerably difficult’, and ‘extremely difficult’ were increased in accordance with progression of ADL disability level

### Associations between the GLFS-25 levels and degree of difficulty on daily activities

**Fig. 2:**
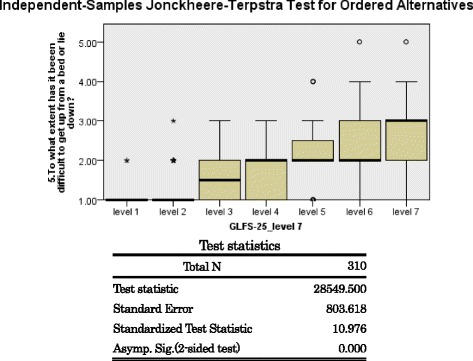
An example result of Jonckheere-Terpstra test on question ‘To what extent has it been difficult to get up from a bed or lie down?’ Hypothesis that the distribution of response items is the same across categories of GLFS-25 level was rejected. The grade of difficulty in individual daily activity increased in accordance with the deterioration of ADL disability

Jonckheere-Terpstra test rejected the null hypothesis that degrees of difficulty were equal between the GLFS-25 levels by *p* value <0.001 for 19 items regarding daily activities, which revealed significant aggregating trend between GLFS-25 levels on difficulty in doing daily activities (Fig. 2). It was revealed that the grade of difficulty in individual daily activity increased in accordance with the deterioration of ADL disability.

### Frequency of poor response ratio (PRR) to questions regarding daily activities (Table 4)

**Table 4:** Poor response ratios for daily activities by the GLFS-25 levels

Activities	GLFS level	Level 1	Level 2	Level 3	Level 4	Level 5	Level 6	Level 7	Total
	Ranges	−6	7–15	16–23	24–32	33–40	41–49	50-	0–73
(Question Items)	N	36	87	68	39	31	27	22	311
21.join sports activity	social activity	5.6	28.7	58.8	76.9	90.3	100.0	100.0^3^	55.7
23.join social activities	social activity	5.6	18.4	26.5	51.3	67.7	66.7	77.3^2^	36.0
13.walk briskly	locomotion	0.0	10.3	39.7	66.7	87.1	92.6	100.0^3^	43.3
12.go up & down stairs	locomotion	0.0	10.3	22.1	53.8	77.4	81.5	95.5^3^	35.7
20.heavy housework	housework	0.0	11.5	22.1	53.8	64.5	77.8	100.0^3^	34.7
15.walk a long distance	locomotion	0.0	23.0	44.1	48.7	67.7	85.2	95.5^3^	42.7
17.carry heavy objects	housework	0.0	9.2	25.0	43.6	48.4	59.3	95.5^3^	30.3
18.use public transportation	locomotion	0.0	2.3	7.4	23.1	45.2	55.6	95.5^3^	21.0
6.stand up from chair	mobility	0.0	0.0	7.4	10.3	19.4	44.4	77.3^2^	14.0
5.get up from bed	mobility	0.0	1.1	7.4	12.8	25.8	33.3	59.1^2^	13.1
19.simple tasks	housework	0.0	0.0	4.4	7.7	12.9	25.9	77.3^2^	10.8
16.visit neighbors	keep company	0.0	0.0	0.0	10.3	12.9	37.0	59.1^2^	9.9
14.keep neat	self-care	0.0	0.0	0.0	0.0	9.7	14.8	54.5^2^	6.1
22.meet friends	keep company	0.0	2.3	13.2	28.2	45.2	29.6	50.0^1^	17.8
11.wash body	self-care	0.0	0.0	0.0	7.7	12.9	29.6	50.0^1^	8.3
9.put on pants	self-care	0.0	0.0	0.0	7.7	12.9	37.0	45.5^1^	8.6
7.walk indoors	mobility	0.0	0.0	1.5	5.1	12.9	18.5	40.9^1^	6.7
8.put on shirts	self-care	0.0	2.3	1.5	0.0	9.7	14.8	31.8^1^	5.4
10.use toilet	self-care	0.0	0.0	0.0	2.6	3.2	7.4	36.4^1^	3.8
number of the items with PRR 50%<		0	0	1	5	6	8	15	

The frequency of activity limitations was increased level by level in accordance with the progression of ADL disabilities except ‘put on shirts’ and ‘use toilet’

Aligned PRR on all participants in descending order

﻿Nineteen items in the level 7 divided into 3 groups which are indicated by superscript 1-3

PRR corresponded to degree of difficulty on activities within GLFS-25 level and patterns of progression of disability.

In level 1, the 0% PRR was found in all but 2 question items (‘join sports activity’, ‘join social activity’). In level 2, the 10% < PRR in 6 (‘join sports activity’, ‘walk a long distance’, ‘join social activities’, ‘heavy housework’, ‘walk briskly’, ‘go up & down stairs’). In level 3, the 20% < PRR in 7 (those 10% < in level 2 plus ‘carry heavy object’). In level 4, the 20% < PRR in 9 (those 20% < in level 3 plus ‘use public transportation’, ‘meet friends’). Its 50% < PRRs were more than half of 8 activities. In level 5, the 50% < PRR in 6 (those 50% in level 4 plus ‘walk a long distance’). In level 6, the 50% < PRR in 8 (those 50% < in level 6 plus ‘carry heavy object’, ‘use public transportation’). In level 7, the 50% < PRR in all but 4 items (‘put on pants’, ‘walk indoors’, ‘use toilet’, and ‘put on shirts’). The 100% PRR were ‘sports activity’, ‘walk briskly’ and ‘heavy housework’.

We identified 3 groups of activities characterized by the level of PRR in level 7. (Table 4).

Group 1: Activities with mild difficulty: 6 activities (4 regarding self-care, 1 mobility, and 1 interpersonal interaction). Less than half of the participants reported difficulties for the 6 activities.

Group 2: Activities with moderate difficulty: 6 activities: 2 regarding mobility, 1 domestic life, 1 social life and 1 interpersonal interaction. More than half (50–80%) of the participants reported difficulties for the 6 activities.

Group 3: Activities with severe difficulty: 7 activities: 4 regarding mobility, 2 domestic life, 1 social life. Almost all of the participants reported difficulties for the 7 activities.

Activities included in group 1 were considered the easiest to perform, and those in included in group 3 the hardest. The groups of activities were suggestive of hierarchical order of activity limitations.

These present results showed both increasing number of activity limitation and progression of severity in difficulty in activities were significantly related to the levels of ADL disability. The results also showed difficulties in mobility appeared in less severe level, difficulties in domestic and social life appeared in moderately severe level, and difficulties in self-care appeared in advanced level.

## Discussion

Participants of this study were elderly people with locomotive disorders and they reported difficulties in basic and instrumental ADL. Mean GLFS-25 score (23.0, and SD 15.8) indicated participants were satisfied criteria for identifying persons with LoS. The participants were considered to be fitted to the people who were in the condition corresponded to the concept of Locomotive syndrome [7].

Disability in ADL is an adverse outcome of frailty that places a burden on elderly people, families, care providers, and the care system [29]. Frailty is defined as a state of increased vulnerability to poor resolution of homeostasis after stress or events [30]. Frailty is a heterogeneous condition consisting of symptoms which do not attribute specifically to a certain organ.

Physical frailty and cognitive frailty were defined as special forms of frailty. Physical frailty is a special form of frailty defined as a medical syndrome with multiple causes and contributors that is characterized by diminished strength, endurance, and reduced physiologic function that increases an individual’s vulnerability for developing increased dependency and/or death [31]. Another specific form of frailty, cognitive frailty, was proposed as a new clinical condition with coexisting physical frailty and cognitive impairment in non-demented older subjects [32].

LoS is one of the potential causes of physical frailty and locomotive organ-targeted state which developed to aim at controlling the increase in elderly people requiring assistance from others (LTCI care needs). The reasons why we newly introduce LoS as specific form of physical frailty are association of pain problems and multiple involvements in locomotive organs requiring medical treatments or care supports.

Although physical impairments and declined motor ability were proved to relate to the GLFS-25 score, examination on the characteristics of disability (numbers and severity of activity limitations, corresponding to the GLFS-25 total score) was not sufficient. Studies to determine risk factors or predictors of LoS were conducted using the GLFS-25 [13–20]. However those previous studies did not provide insight into ADL disability. Identification of factors related to severity of ADL disability is necessary to develop intervention program to prevent or slow down aggravation of ADL disability.

The results from present study showed significant relationships of disability level with the number and severity of activity limitation. The findings suggested that aggravation of ADL disability level is associated with increase of numbers of activity limitations and deterioration of difficulty in executing activities.

Iwaya, Doi and Akai [20] reported that the prevalence of persons reported difficulty was high in walking related activities (‘walk briskly’, ‘walk a long distance’, ‘go up and down stairs’) and low in self-care activities (‘put on pants’, ‘use toilet’, ‘take a bath’) in people with mild level of ADL disability, and the prevalence was high in both activities in advanced level of disability. In the present study, we suggested hierarchical order of activity limitations in progression of disability. The hierarchical order was consistent with those reported previous articles [33–35].

Older adults who are disabled can recover from a disabled state [36]. To reduce numbers of activity limitation and/or ameliorate difficulty in daily activities may contribute recovery from disability.

In mild disability level (level 2–4), frequency of activity limitations was high in mobility related activities, and low in self-care related activities. In the advanced level (level 5–7) frequency of activity limitations was high in both mobility and self-care related activities. These findings suggested difficulties in mobility related activities, particularly walking related activities, may be ahead of ADL disability in elderly people with locomotive disorders. Lawrence and Jett [37] described that lower extremity functional limitation was a stronger predictor of disability than upper extremity. Shinkai et al. [38] reported walking speed as a good predictor for the onset of functional dependence, defined as a new disability in one or more of the five basic activities of daily living, or death of a subject who had shown no disability at the previous follow-up in a Japanese rural community population.

Findings from present study revealed that people with mild ADL disability experienced difficulties of mild degree in mobility activity and people with advanced ADL disability experienced difficulties of moderate to severe degree in mobility activity and difficulties of mild degree of self-care activities. According to the findings, we assume that physical exercise to restore walking capacity such as aerobic exercise or strengthening exercise of lower leg muscles are efficient to prevent deterioration of ADL disability for people in level 2 and 3. Interventions to reduce difficulties in self-care activities may benefit people in level 4 and over. Those interventions include not only physical exercise such as ROM exercise, stretching, but using aids or devices, personal assistance and accommodation of living conditions.

Two types of disability are distinguished, namely progressive and catastrophic disability [39]. Progressive disability develops with older age in association with underlying diseases, comorbidity and frailty and resulted from physical functional decline. Disabilities develop abruptly (sudden onset) or insidiously and progress step by step. Catastrophic disability is the result of an acute clinical event such as hip fracture or stroke. In elderly people disability may begin abruptly in cases of hip fractures, may progress slowly such as in cases of LoS and may suddenly deteriorate following accidental events such as fall or pneumonia or death of spouse.

Progressive disability may take years to make elderly people disabled. There may be people without functional limitations who have impairment, and people without disability who have functional limitations. Identification of people without disability who have functional limitations and people without functional limitations who have impairments allows for appropriate approach at different points in the disablement pathway [40]. Similarly, identification of people with mild disability and with severe disability allows for appropriate approach at different points in deterioration process of disability.

### Limitation of the present study

Because this study used cross-sectional data, there are several limitations. Findings that number and severity of activity limitations significantly related to severity of disability are suggestive of pattern of progression of disability. Disabilities develop with time and the processes are dynamic by nature. The applicability of the patterns of disability progression should be verified in longitudinal survey data. The hierarchic model presented in this article is also hypothetical and need to be tested in a longitudinal study.

Concept of LoS is not satisfactory operationalized and characteristics of LoS are not well described at present time. Further studies on disablement process describing progression from locomotive organ disorders (degenerative arthritis, osteoporosis, sarcopenia) to impairment (pain on lumbar spine and lower extremity, ROM limitation, muscle weakness) to functional limitation (slow walking speed, weak muscle strength, reduced flexibility) and disability (difficulties experienced during doing familiar tasks) are needed to develop strategies for prevention and restoration of disabilities resulting from locomotive organ disorders.

## Conclusions

Participants with higher GLFS-25 score reported larger numbers of activity limitations of greater degree of difficulty. High GLFS-25 score represented high degree of ADL disability. Disability may deteriorate in accordance with increase of numbers and aggravation of activity limitation. Activity limitation may occur in the following order: sports activity, walking, transferring, and self-care.

## Abbreviations

% YAM: % young adults mean
ADL: Activity of daily living
DEXA: Dual-energy X-ray absorptiometry
GLFS −25: Geriatric Locomotive Function Scale – 25
LoS: Locomotive syndrome
LTCI: Long-Term Care Insurance
PRR: Poor response ratio
QCT: Quantitative computed tomography
ROM: Range of motion
SD: Standard deviation
